# Neuroprotective Properties of Picroside II in a Rat Model of Focal Cerebral Ischemia

**DOI:** 10.3390/ijms11114580

**Published:** 2010-11-16

**Authors:** Qin Li, Zhen Li, Xin-ying Xu, Yun-liang Guo, Fang Du

**Affiliations:** 1 Institute of Cerebrovascular Diseases, Affiliated Hospital of Qingdao University Medical College, Qingdao Shandong 266003, China; E-Mails: liqin1015@yahoo.com.cn (Q.L.); leerushui@yahoo.com.cn (Z.L.); xuxinying66@126.com (X.X.); dufang1999@163.com (F.D.); 2 Emergency Centre of Yantai Yuhuangding Hospital, Yantai Shandong, 264000, China

**Keywords:** picroside II, cerebral ischemia, reperfusion injury, caspase-3, PARP, apoptosis, rats

## Abstract

The aim of this study was to explore the effect of picroside II on neuronal apoptosis and the expression of caspase-3 and poly ADP-ribose polymerase (PARP) following middle cerebral artery occlusion/reperfusion in male Wistar rats. Picroside II (10 mg/kg) was administered intravenously into the tail vein of the animals. The neurological function deficits were evaluated with the Bederson’s test and the cerebral infarction volume was visualized with tetrazolium chloride (TTC) staining. The apoptotic cells were counted by *in situ* terminal deoxynucleotidyl transferase-mediated biotinylated deoxyuridine triphosphate nick end labeling (TUNEL) assay. The immunohistochemistry stain and enzyme linked immunosorbent assay (ELISA) was used to determine the expressions of caspase-3 and PARP in brain tissue. The results indicated that rats in the control group showed neurological function deficit and cerebral infarction in ischemic hemisphere after two hours ischemia followed by 22 hours reperfusion. Caspase-3 and PARP expressions were also profound in the cortex, the striatum and the hippocampus, along with increased apoptotic cells in this group. Bederson’s score, infarction volume, and expressions of caspase-3 and PARP, as well as apoptosis in the treatment group were, however, significantly decreased compared to those in the control group indicating that intravenous treatment with picroside II might be beneficial to inhibit neuronal apoptosis and, thus, to improve the neurological function of rats upon cerebral ischemia reperfusion injury.

## 1. Introduction

Studies have shown that the caspase-family is the promoter and implementer of apoptosis in mammalian cells, among which, caspase-3 is the most critical downstream apoptosis protease in the caspase cascade “waterfall” [1]. A variety of extracellular signals activate caspase-8 through Fas receptor pathway and caspase-9 via mitochondrial cytochrome C in cerebral ischemia reperfusion injury. Activation of caspase-8 and caspase-9 then promote caspase-3, which in turn hydrolyzes cell-specific proteins, and poly ADP ribose polymerase (PARP), thereby inducing apoptosis [2,3].

**Figure 1.**
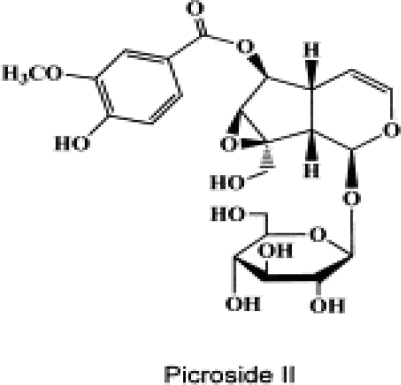
The chemical structure of picroside II (β-d-glucopyranoside, 1a,1b,2,5a,6,6a-hexahydro-6-[(4-hydroxy-3-methoxybenzoyl)oxy]-1a(hydroxymethyl)oxireno Cyclopenta [1,2-c]pyran-2-yl) [7].

The plant *Picrorhiza scrophulariiflora* (*Scrophulariaceae*) grows in a high altitude in certain regions of Tibet and China. The roots of this plant are used in traditional Chinese medicine for a number of conditions [4]. Extracts of the roots contain various terpenoids as well as glycosides [5,6]. One of the main active constituents of the extract is picroside II, which is an iridoid glucoside (Figure 1).

Current research on picroside II is focused on its neuroprotective [8], antiapoptotic, anticholestatic, antioxidant, anti-inflammatory, immunemodulating activities [9–11]. Some studies implicate that picroside II protects hepatocytes against injury and counteracts apoptosis through maintaining the integrity of the mitochondria membrane, enhancing the activity of ATPase in mitochondria, thereby modulating the balance of the cell energy metabolism [12,13]. Li’s and Tao’s data show that picroside II can reduce H_2_O_2_-induced PC12 cell damage and improve cell survival [14–16]. Experiments on animal models indicate that the picroside extract could inhibit apoptosis in ischemic penumbra in rats following middle cerebral artery occlusion and reperfusion (MCAO/R) [17]. Our previous experiment showed that picroside II inhibited the expressions of NFκB and IκB following MCAO/R in rats [18]. The present study aims to explore the properties of picroside II in rat model of focal cerebral ischemia.

## 2. Results and Discussion

### 2.1. Neurobehavioral Deficit Score

There was no neurobehavioral dysfunction symptom in rats of the sham-operation group, whose Bederson’s score was 0. After cerebral ischemic reperfusion injury, all animals showed neurological defects. The Bederson’s scores in the treatment group were obviously lower than that in the control group (*t* = 5.21, *P* < 0.05). See Table 1.

### 2.2. Volume of Cerebral Infarction

**Figure 2.**
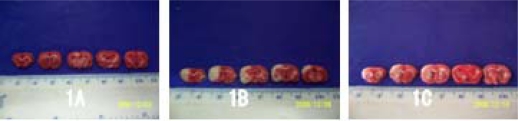
Cerebral infarction volume shown by TTC stain. The normal brain tissue appeared uniformly red in the sham-operation group (**A**), while the infarction region showed white in the control group (**B**) and treatment group (**C**).

By TTC stain, no ischemia infarction was shown in the brain slices of the sham-operation group, while infarction lesion appeared in all the experimental rats after cerebral ischemic reperfusion injury. The volume of cerebral infarction in the treatment group were significantly lower than that in the control group (*t* = 4.19, *P* < 0.05); see Table 1 and Figure 2.

### 2.3. Neuronal Apoptosis

**Figure 3.**
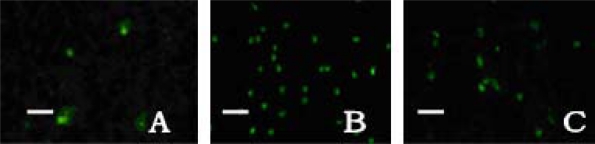
Apoptotic cells in cortex shown by TUNEL × 200. Only a few apoptotic cells in the sham-operation group (**A**), increased in the control group (**B**) and decreased in the treatment group (**C**) significantly. Bar 50 μm.

A few apoptotic cells were scattered in the cortex and the striatum in the sham-operation group. Apoptotic cells were significantly increased in the control group. As we expected, the amount of apoptosis in the treatment group was low compared to those found in the cortex (*F* = 194.10, *q* = 4.60–26.10, *P* < 0.01), striatum (*F* = 167.94, *q* = 5.13–24.57, *P* < 0.01) and hippocampus (*F* = 230.60, *q* = 6.51–28.95, *P* < 0.01) of the control group; see Table 2 and Figure 3.

### 2.4. Caspase-3 Expression

**Figure 4.**
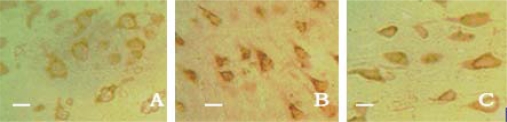
Caspase-3 positive cells in cortex shown by immunohistochemical assay × 400. The expression of Caspase-3 was very weak in the sham-operation group (**A**), increased in the control group (**B**) and decreased in the treatment group (**C**) significantly. Bar 25 μm.

With the help of immunohistochemistry, we found that there were no region differences between the cortex, the striatum and the hippocampus. Thus, we calculated the absorbance values (*A*) in four confirmed views instead of the random ones in each brain slice. Only a small number of caspase-3 positive cells with light yellow granules could be seen in the cortex, the striatum and the hippocampus in sham-operation group rats, and its expression was very weak. Caspase-3 expression in control group was significantly elevated along with the increased *A* value as compared with the sham-operation group rats. After the drug administration, the *A* values in the treatment group were obviously decreased in contrast to the control group rats in cortex (*F* = 99.95, *q* = 7.43–19.79, *P* < 0.01), striatum (*F* = 121.18, *q* = 7.49–21.68, *P* < 0.01) and hippocampus (*F* = 107.11, *q* = 8.02–20.54, *P* < 0.01). It is suggested that picroside II could inhibit the expression of caspase-3 protein and play neuroprotective effects (Table 3 and Figure 4).

### 2.5. PARP Expression

**Figure 5.**
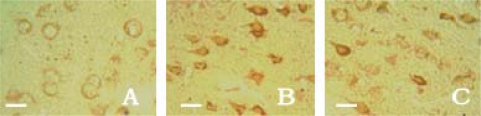
PARP positive cells in cortex shown by immunohistochemical assay × 400. The expression of PARP was very weak in the sham-operation group (**A**), increased in control group (**B**) and decreased in treatment group (**C**) significantly. Bar 25 μm.

Weak PARP expression was shown in the cortex, the striatum and the hippocampus in sham-operation group rats. The number of PARP-positive cells rapidly increased, and the *A* value was significantly higher than that in the sham-operation group. The expression of PARP both in the treatment group were apparently lower than that in the control group in cortex (*F* = 142.48, *q* = 9.23–23.68, *P* < 0.01), striatum (*F* = 137.25, *q* = 9.37–23.28, *P* < 0.01) and hippocampus (*F* = 141.94, *q* = 8.66–23.56, *P* < 0.01) shown in Table 4 and Figure 5.

### 2.6. The Concentration of Caspase-3 and PARP in Brain Tissue

The concentrations of caspase-3 and PARP were low in the brain tissue of the sham-operation group rats, and increased significantly in the control group rats. In the treatment group, the concentration of caspase-3 (*F* = 86.25, *q* = 8.11–18.52, *P* < 0.01) and PARP (*F* = 108.11, *q* = 9.33–20.76, *P* < 0.01) were obviously lower than those in the control group (Table 5).

### 2.7. Discussion

Among various mechanisms, the modulation of caspases expression plays a critical role for the common pathway of apoptosis. Caspase-3 is a key member of executioner caspases. Using rat MCAO/R models, Yang *et al*. [19] found the expression of caspase-3 enhanced with the increased cell apoptosis in cerebral cortex at 1h ischemia and 24 h reperfusion. Xu’s studies [20] have shown that caspase-3 activity in the striatum and the dorsolateral cortex was significantly increased upon 50 min ischemia followed by 24 h reperfusion in MCAO/R rats. Apoptosis is significantly increased in ischemic central and penumbra area. Puerarin, a major isoflavonoid derived from the Chinese medical herb kudzu root can significantly reduce apoptosis and inhibit caspase-3 activity in dorsolateral cortex in the rat brain of 2 h cerebral ischemia followed by 46 h reperfusion [21]. The research results of Tang *et al*. [22] indicated that alkaloid, glycoside, polysaccharide and aglycone from Buyang huanwu decoction (BYHWD) could relieve the inflammatory reaction occurring after cerebral ischemia/reperfusion by inhibiting Caspase-1 expression to decrease production of inflammatory cytokine. PARP is a kind of protease with catalytic activity of poly ADP ribosylation (PAR) in eukaryotic cell, and plays a role in the maintenance of chromosome stability, DNA damage recovery, gene transcription, cell growth, death and apoptosis. Moroni [3] suggests that the cerebral ischemia caused oxidative DNA damage and PARP expression increased in neurons. With the duration of ischemia or reperfusion and the accumulation off DNA damage, PARP gene expression is also heavier. The recovery ability of PARP in cell DNA damage depends on the intracellular coenzyme I (dihydrouracil dehydrogenase, NAD^+^) level. Under conditions of adequate NAD^+^, the increasing PARP activity is conducive to DNA recovery and promotes neuron survival [23]. Chaitanya *et al*. [24] established ischemia 3 h and reperfusion 1–24 h rat models and found that the expression of caspase-3 increases in cerebral cortex with the duration of ischemic time, caspase-3 hastens PARP degradation, resulting in neuronal apoptosis. Li *et al*. reported that picroside II could have a neuroprotective effect by inhibiting the apoptosis and the expressions of NFκB and IκB following MCAO/R in rats [18]. It also reduced brain tissue edema and the aquaporin-4 (AQP-4) expression, and established the best therapeutic time window was one hour after cerebral ischemic reperfusion [25]. The present experiments showed that the expressions of caspase-3 and PARP elevated in the cortex, the striatum and the hippocampus in rats after cerebral ischemia 2 h and reperfusion 22 h, together with the increased apoptosis cells and infarction volume, as well as aggravated neurobehavioral dysfunction. Via the tail vein administration of picroside II, the expression of caspase-3 is weaker than that in the control group in the cortex, the striatum and the hippocampus. The cerebral infarction size was obviously reduced, and neurobehavioral function significantly improved in the treatment groups.

These data suggest that picroside II may inhibit caspase-3 expression and reduce PARP degradation after cerebral ischemic injury, which may contribute to the maintenance of NAD^+^ level in penumbra, thereby enabling PARP to utilize residual energy for the reparation of neuronal damage. Inhibiting apoptosis ultimately leads to improved neurobehavioral outcome.

## 3. Experimental Section

### 3.1. Animal Model of MCAO/R

The total of 60 adult female Wistar rats, weight 230–250 g, SPF grade, were granted by Qingdao Laboratory Animal Center (SCXK (LU) 20070010). The local legislation for ethics of experiment on animals and guidelines for the care and use of laboratory animals were followed in all animal procedures [26]. All animals were given time to adapt to the laboratory environment, allowed free access to food and water in a temperature and humidity-controlled housing with natural illumination for a week, and fasted 12 h before operation. Fifteen rats were randomly selected as a sham-operation group, and the remaining 45 rats were subjected to the experimental middle cerebral artery ischemia for 2h followed by 22 h of reperfusion. Ischemia was induced by intraluminal monofilament suture in the left external-internal carotid artery [27]. Core body temperature was monitored with a rectal probe and maintained at 36–37 °C using a homeothermic blanket control unit (Qingdao Apparatus, China) during and after the surgery operation. Rats in sham-operation group were subjected to the same surgical procedure but the monofilament was advanced only about 10 mm and immediately withdrawn. Thirty successful MCAO/R rat models were internalized into the experiment group (15 unsuccessful animals were removed), and then randomly divided into a control group and a treatment group consisting of 15 rats in each group.

### 3.2. Intervention Study

Picroside II was obtained from Kui Qing, Tianjin Medical Technology Co., Ltd., (CAS No: 39012-20-9, purity > 98%). It was diluted into 1% solution with 1 M PBS sodium. According to Xiao’s report [28], the rats in the treatment group were administrated picroside II (10 mg/kg) 250 μL via tail vein at the end of ischemic 2 h before reperfusion 22 h with a micro-syringe, while those in the control group and the sham-operation groups were simultaneously injected 1 M PBS 250 μL.

### 3.3. Neurobehavioral Dysfunction Score

All animals were scored at ischemia 2 h reperfusion 22 h by an investigator who was blinded to the experiment according to the standard of Bederson’s test [29]. 0 score: no neurological functional impairment; 1 score: any part of forepaw flexed (positive for tail suspension test) without other abnormal sign; 2 scores: lateral pushing resistance ability decreased (positive for lateral pushing experiment), accompanied with forepaw flexion without circling tendency; 3 scores: same behaviors as those for 2 scores, in addition to spontaneous rotation (circling around paralyzed limbs during free activity). The higher the score is, the worse the neurobehavioral dysfunction appears, and *vice versa*.

### 3.4. TTC Staining

To determine the infarction volume, five rats in each group were decapitated at ischemia 2 h reperfusion 22 h after MCAO/R. The brain tissue was removed and successively sliced into 2.0 mm thick coronal sections. The total of five brain slices were incubated in 2% TTC solution for 10 min at 37 °C and then transferred into 4% formaldehyde solution for fixation. Normal brain tissue appeared uniform red while the infarction region showed white. The infarction volumes were calculated in a blinded manner with Adobe PhotoShop CS analysis system. The data were expressed as the percentage of the infarction volume/the ipsilateral hemisphere volume (%) at the coronal section of optic chiasma.

### 3.5. Neuronal Apoptosis Assay

At ischemia 2 h reperfusion 22 h after MCAO/R, five rats in each group were deeply anesthetized by 10% chloral hydrate (300 mg/kg), reperfused with sodium chloride and 4% formaldehyde 200 mL from the heart into the aorta, and then decapitated at given time. Brain samples were chosen from frontal fontanelle 2 mm to occipital fontanelle 4 mm by a stereotaxic point, post-fixed in 4% formaldehyde for 2 h, dehydrated in alcohol gradually, hyalinized by dimethylbenzene, embedded in paraffin, then sectioned at a thickness of 5 μm, adhered to the sections prepared with poly-l-lysine, and finally stored at 4 °C. To detect cell apoptosis, TUNEL staining was performed according to the protocol of DendEnd fluorometric TUNEL detection system (Santa Cruz Co. Ltd.). In each rat, four coronal paraffin sections as described above were deparaffinaged by dimethylbenzene, hydrated by gradient ethanol and washed by distilled water. To some sections DNase I at a dose of 1 μg/mL was added and were regarded as the positive control sample, and those treated without TdT were the negative ones. Under a 400-fold immunofluorescent microscope (wavelength 488 nm), the apoptosis cells appeared fluorescent yellow-green in the nucleus, and averaged in four random views of the cortex, and striatum, respectively.

### 3.6. Immunohistochemical Staining

Rabbit anti-rat Caspase-3 and PARP moloclonal antibody, SABC immunohistochemistry kit, DAB dye were purchased from Boster Biological Company, Wuhan, China. Paraffin-embedded sections were deparaffinaged in dimethylbenzene, hydrated successively in gradient ethanol, and antigen was restored twice in a microwave oven. Immunohistochemical procedures were performed strictly according to the manufacturer’s guidelines. Under a microscope, those with brown granules in cytoplasm were considered as positive cells. And the slides with the addition of 0.01 mmol/L PBS (containing 1:200 non-immunity animal serum), instead of primary antibody, showed no response. Four serial sections were chosen from each experimental rat, and four views of the cortex, striatum hippocampus in each section were observed randomly under a 400-fold fluorescent microscope. Absorbance value (A) of each view was detected by a LEICA Qwin microgramme analytical system (Leica Company).

### 3.7. Enzyme Linked Immunosorbent Assay (ELISA)

Rat caspase-3 and PARP ELISA kits were purchased from Blue Gene Co. Ltd. Five rats in each group were deeply anesthetized and decapitated at given time after MCAO/R. At ischemia 2 h reperfusion 22 h after MCAO/R, the ischemic hemisphere tissues (0.5 g) from rats in all groups were quickly removed and ground fully into brain tissue homogenate. Normal sodium 500 μL was then added, mixed well and centrifuged for 10 minutes at 12,000 rpm. The upper limpid liquid was collected and stored at −20 °C (to avoid repeated freeze-thaw cycles). All standards were prepared before starting assay procedures. Firstly, we secured the desired number of coated wells in the holder and added 50 μL of standards or samples to the appropriate well of the antibody pre-coated microtiter plate. 100 μL of conjugate was then added to each well, mixed thoroughly, covered and incubated for 1 h at 37 °C. The microtiter plate was washed five times using distilled or de-ionized water; 50 μL of substrate A and B was added to each well, covered and incubated for 15 minutes at 25 °C. Finally, 50 μL of stop solution was added to each well, thoroughly mixed and the mean absorbance value at 450 nm for each set of reference standards and samples was calculated. Calculation of results: The average A450 value for each standard, control and test sample was divided by the average A450 of the standard 0 and multiplied by 100 to obtain %B/B_0_ for each sample. A standard curve was prepared by plotting the average absorbance or the %B/B0 value of each standard (on the y axis) *versus* the corresponding concentrations of the standards (on the x axis) on linear-log graph paper or logit-log graph paper. According to the B/B0 value of the samples, the concentration of the samples can be determined from the standard curve. The sensitivity by this assay is 1.0 ng/mL.

### 3.8. Statistical Analysis

SPSS11.5 software was used for statistical analysis. Data were expressed as mean ± standard error (*x̄* ± s). Multi-group comparison was made by analysis of variance (ANOVA) and Student’s test, and two-group comparison by *t*-test. Values were considered to be significant when *P* was less than 0.05.

## 4. Conclusions

This study suggested that picroside II might reduce the expressions of Caspase-3 and PARP to inhibit the neuronal apoptosis induced by cerebral ischemia reperfusion injury and improve the neurological function of rats.

## Figures and Tables

**Table 1.** Neurobehavioral deficit score and infarct volume (*x̄* ± s).

**Groups**	**n**	**Neurological function scores**	**Infarction volume**
Sham-operation group	5	0.00 ± 0.00	0.00 ± 0.00
Control group	5	2.16 ± 0.28Δ	77.32 ± 3.06Δ
Treatment group	5	1.27 ± 0.26*	68.73 ± 3.46*

Δ *t* = 5.21, *P* < 0.05 *vs.* the sham-operation group;

* *t* = 4.19, *P* < 0.05 *vs.* the control group.

**Table 2.** The number of apoptotic cells in different brain regions (*x̄* ± s).

**Groups**	**n**	**Cortex**	**Striatum**	**Hippocampus**
Sham-operation group	5	4.53 ± 1.13	3.79 ± 1.36	3.67 ± 1.15
Control group	5	67.62 ± 8.25Δ	53.30 ± 6.26Δ	41.30 ± 4.24
Treatment group	5	15.64 ± 4.28*	14.12 ± 4.46*	12.13 ± 2.46*

Δ *P* < 0.05 *vs.* the sham-operation group;

* *P* < 0.05 *vs.* the control group.

**Table 3.** The number of Caspase-3 positive cells (*x̄* ± s).

**Groups**	**n**	**Cortex**	**Striatum**	**Hippocampus**
Sham-operation group	5	5.25 ± 1.70	4.25 ± 1.95	3.75 ± 1.69
Control group	5	33.4 ± 4.07Δ	32.80 ± 3.77Δ	28.00 ± 3.58 Δ
Treatment group	5	15.82 ± 3.30*	14.12 ± 2.83*	13.22 ± 2.29*

Δ *P* < 0.05 *vs.* the sham-operation group;

* *P* < 0.05 *vs.* the control group.

**Table 4.** The number of PARP positive cells (*x̄* ± s).

**Groups**	**n**	**Cortex**	**Striatum**	**Hippocampus**
Sham-operation group	5	4.34 ± 1.24	3.72 ± 1.18	3.28 ± 1.16
Control group	5	34.25 ± 4.21Δ	31.75 ± 3.70Δ	27.50 ± 3.29 Δ
Treatment group	5	16.00 ± 2.16*	15.00 ± 2.58*	12.20 ± 1.92*

Δ *P* < 0.05 *vs.* the sham-operation group;

* *P* < 0.05 *vs.* the control group.

**Table 5.** The concentration in brain tissue of caspase-3 and PARP (*x̄* ± s).

**Sham-operation group**	**n**	**Caspase-3**	**PARP**
Control group	5	12.35 ± 2.21	12.24 ± 2.23
Treatment group	5	54.23 ± 7.22Δ	48.12 ± 5.17Δ
Sham-operation group	5	30.45 ± 4.44*	28.36 ± 3.62*

Δ *P* < 0.05 *vs.* the sham-operation group;

* *P* < 0.05 *vs.* the control group.

## References

[1] Cho BB, Toledo-Pereyra LH (2008). Caspase-independent programmed cell death following ischemic stroke. J. Invest. Surg.

[2] Broughton BR, Reutens DC, Sobey CG (2009). Apoptotic mechanisms after cerebral ischemia. Stroke.

[3] Moroni F (2008). Poly (ADP-ribose) polymerase-1 (PARP-1) and postischemic brain damage. Curr. Opin. Pharmacol.

[4] Li JX, Li P, Tezuka Y, Namba T, Kadota S (1998). Three phenylethanoid glycosides and an iridoid glycoside from Picrorhiza scrophulariiflora. Phytochemistry.

[5] Wang DQ, He ZD, Feng BS, Yang CR (1993). Chemical constituents from Picrorhiza scrophulariiflora. Acta Botanica Yunnanica.

[6] Zou LC, Zhu TF, Xiang H, Yu L, Yan ZH, Gan SC, Wang DC, Zeng S, Deng XM (2008). New secoiridoid glycosides from the roots of Picrorhiza Scrophulariiflora. Molecules.

[7] Li P, Matsunaga K, Yamakuni T, Ohizumi Y (2000). Potentiation of nerve growth factor-action by picrosides I and II, natural iridoids, in PC12D cells. Eur. J. Pharmacol.

[8] Li T, Liu JW, Zhang XD, Guo MC, Ji G (2007). The neuroprotective effect of picroside II from hu-huang-lian against oxidative stress. Am. J. Chin. Med.

[9] Cao Y, Liu JW, Yu YJ, Zheng PY, Zhang XD, Li T, Guo MC (2007). Synergistic protective effect of picroside II and NGF on P12 cells against oxidative stress induced by H_2_O_2_. Pharmacol. Rep.

[10] Smit HF, Kroes BH, van den Berg AJ, van der Wal D, van den Worm E, Beukelman CJ, van Dijk H, Labadie RP (2000). Immunomodulatory and anti-inflammatory activity of Picrorhiza scrophulariiflora. J. Ethnopharmacol.

[11] He LJ, Liang M, Hou FF, Guo ZJ, Xie D, Zhang X (2009). Ethanol extraction of Picrorhiza scrophulariiflora prevents renal injury in experimental diabetes via anti-inflammation action. J. Endocrinol.

[12] Gao H, Zhou YW (2005). Inhibitory effect of picroside II on hepatocyte apoptosis. Acta Pharmacol Sin.

[13] Gao H, Zhou YW (2005). Anti-lipid peroxidation and protection of liver mitochondria against injuries by picroside II. World J. Gastroenterol.

[14] Li P, Matsunaga K, Yamakuni T, Ohizumi Y (2000). Potentiation of nerve growth factor-action by picrosides I and II, natural iridoids, in PC12D cells. Eur. J. Pharmacol.

[15] Li P, Matsunaga K, Yamakuni T, Ohizumi Y (2002). Picrosides Iand II, selective enhancers of the mitogen-activated protein kinase-dependent signaling pathway in the action of neuritogenic substances on PC12D cells. Life Sci.

[16] Tao YW, Liu JW, Wei DZ, Su W, Zhou WY (2003). Protective effect of Picroside II on the damage of PC12 cells *in vitro*. Chin J Clin Pharmacol Ther.

[17] Yin JJ, Zhang W, Du F (2005). Effects of extraction of Huhuanglian on apoptosis and Bcl-2 gene in penumbra area in ischemia reperfusion rats. Shandong J Tradit Chin Med.

[18] Li Z, Xu XY, Shen W, Guo YL (2010). The interferring effects of picroside II on the expressions of NF-κB and I-κB following cerebral ischemia reperfusion injury in rats. Chin Pharmacol Bull.

[19] Yang X, Zhang L, Jiang S, Gong P, Zeng F (2009). Effect of dauricine on apoptosis and expression of apoptogenic protein after transient focal cerebral ischemia-reperfusion injury in rats. Chin J Chin Materia Medica.

[20] Xu XH, Chen Y, Zheng XX (2006). Effects of puerarin on neuronal apoptosis induced by cerebral ischemia in rats. Chin Pharm J.

[21] Li H, Deng CQ, Chen BY, Chen RF, Zhang SP, Liang Y (2006). Effects of panax notoginseng saponins on expression of caspase after focal cerebral ischemia reperfusion in rats. Chin Pharmacol Bull.

[22] Tang YH, Li H, Chen BY (2006). Effect of active fraction of buyang huanwu decoction on caspase expression in rats after focal cerebral ischemic reperfusion. Chin. J. Internet. Med.

[23] Liu D, Gharavi R, Pitta M (2009). Nicotinamide prevents NAD^+^ depletion and protects neurons against excitotoxicity and cerebral ischemia: NAD^+^ consumption by SIRT1 may endanger energetically compromised neurons. Neuromol. Med.

[24] Chaitanya GV, Babu PP (2009). Differential PARP cleavage: An indication of heterogeneous forms of cell death and involvement of multiple proteases in the infarct of focal cerebral ischemia in rat. Cell Mol. Neurobiol.

[25] Li Z, Xu XY, Li Q, Zhang MZ, Shen W (2010). Protective mechanisms of picroside II on aquaporin-4 expression in a rat model of cerebral ischemia/reperfusion injury. Neural Regen Res.

[26] (2006). Guidance Suggestion of Caring Laboratory Animals.

[27] Longa EZ, Weinstein PR, Carlson S, Cummins R (1989). Reversible middle cerebral artery occlusion without craniectomy in rats. Stroke.

[28] Xiao YJ, Jiang ZZ, Yao JC, Huang X, Huang JF, Wang T, Zhang LY (2008). Investigation of picrosideII’s impacts on the P450 activities using a cockail method. Chin J Nat Med.

[29] Bederson JB, Pitts LH, Tsuji M, Nishimura MC, Davis RL, Bartkowski H (1986). Rat middle cerebral artery occlusion: Evaluation of the model and development of a neurologic examination. Stroke.

